# Genetic and Genomic Consultation: Are We Ready for Direct-to-Consumer Telegenetics?

**DOI:** 10.3389/fgene.2018.00550

**Published:** 2018-12-03

**Authors:** Li Du, Shmuel I. Becher

**Affiliations:** ^1^Faculty of Law, University of Macau, Macau, China; ^2^School of Accounting and Commercial Law, Victoria University of Wellington, Wellington, New Zealand

**Keywords:** telegenetics, telegenomics, direct-to-consumer, genetic services, risks and benefits, privacy, informed consent

## Abstract

Telegenetics, the application of telemedicine in the context of genetic services, is a growing market. One of the recent developments in this field is the use of direct-to-consumer (DTC) marketing to promote and advertise genetic and genomic consultant services to consumers. Using Google.com, we identified providers that promote their telegenetics services online. By analyzing their websites, we identify and examine key points regarding DTC telegenetics: how are telegenetics services portrayed, how is informed consent obtained, and what protections are offered to clients’ personal health information? We found that the portrayal of a wide range of telegenetics services on providers’ websites is extremely positive. The risks associated with the implementation of telegenetics were rarely mentioned. Consent forms were often unavailable and did not cover all of the relevant information. The measures for protecting clients’ personal health information by telegenetics providers were found to be generally inadequate and weak. We concluded that DTC telegenetics may increase patients’ access to genetic counseling with affordable costs. However, before further developing DTC telegenetics, more research and regulatory improvements are required to guarantee truthful advertising, ensure informed consent, secure personal health data sharing, and warrant adequate privacy protection.

## Introduction

Advancements in genetics and information communication technologies have facilitated the development of telegenetics, a form of telemedicine for genetic services (Mitchell and Demiris, 2005). Telegenetics utilizes technological advancements, such as interactive video and high-speed internet connections, to connect in real time with patients in distant locations (Hilgart et al., 2012). Telegenetics services have therefore become more accessible to broader audiences.

With the increasing demand for genetic services since the early 2000s, telegenetics services have been implemented in the United Kingdom, the United States and other European countries (Gray et al., 2000; Gattas et al., 2001; Lea et al., 2005). Previous research has suggested that these services have received increasing levels of satisfaction from both patients and genetic counselors (Buchanan et al., 2015; Zierhut et al., 2018). Recent studies examining telegenetics services in the United States and Europe have indicated that telegenetics could, and should, become a routine counseling service for the evaluation and diagnoses of certain genetic disorders (Hilgart et al., 2012; Otten et al., 2016; Kubendran et al., 2017).

While genetics is the study of heredity, genomics is a much newer field concerned with the complete genetic information based on sequencing and analysis of the whole human genome. Progress in genomic research and precision medicine has led to genomic data being increasingly integrated into clinical practice to improve the accuracy of disease diagnosis and the efficacy of medical interventions (Mirnezami et al., 2012; Krier et al., 2016; Nakagawa and Fujita, 2018). As a result, telegenetics providers have begun to offer genomic medicine and consulting services through what is termed “telegenomics” (De Castro and Turner, 2017).

Since 2016, the business of telegenomics has already attracted strategic investments from big-name venture capital companies, e.g., GE Ventures and Illumina Ventures (Petrone, 2017; Mack, 2018). This reflects, from yet another perspective, the capital market’s expectations for, and confidence in, the potential of telegenomics. Generally speaking, telegenomics bears a potential promise to transform modern medicine into a more precise and personalized practice, also known as “precision medicine” (Mack, 2018). This, in turn, illustrates the independence and importance of the telegenomics market, as a unique market that offers a distinct service.

One recent development in this important and promising field is the use of direct-to-consumer (DTC) marketing, which seeks to promote and advertise telegenetics or telegenomics services to consumers. Existing research has identified potential regulatory issues related to the availability and use of telemedicine in genetic counseling services (Hilgart et al., 2012; Otten et al., 2016; Vrecar et al., 2017). However, these studies are partial in scope and do not comprehensively examine important regulatory issues for the implementation of telegenomics. In particular, the increase in DTC marketing raises legal and policy challenges which current literature does not sufficiently address. To narrow this gap, this study reviews the websites of DTC telegenetics or telegenomics service providers. Specifically, we focus on informed consent, patients’ privacy, and the use of unsubstantiated positive language.

## Materials and Methods

To identify providers who promote their DTC telegenetics or telegenomics services online we used the Google.com search engine, employing keywords: telegenetics and telegenomics. As of April 2018, we identified ten websites providing DTC telegenetics or telegenomics services. We analyzed these ten websites using a coding framework with 21 items that focused on the following key topics: (1) telegenetics services offered, (2) portrayal of telegenetics, (3) benefits and risks associated with telegenetics, (4) informed consent, and (5) measures for protecting clients’ personal health information.

It is important to clarify that our study focuses on providers that advertise, sell, promote and emphasize their expertise in consulting services. It generally does not include those providers who mainly offer and advertise genetic testing, even if these providers offer a supplementary consulting service (Moscarello et al., 2018). However, we do include one provider, Invitae, that offers both genetic testing and counseling in our dataset. This is because Invitae is the only genetic testing company showing up in our Google search. In particular, Invitae has an independent webpage on genetic counseling services, which reflects the importance Invitae attributes to its counseling services. Consequently, we regard Invitae as a genetic counseling provider that the average consumer is able to find online when searching for telegenetics.

The websites were coded by one of the authors, LD. An independent coder with a specialization in international business law then coded again the ten websites in order to verify the reliability of the coding. We calculated the correlation between the two codes, using Miles and Huberman’s method (Miles and Huberman, 1994), computing the agreement as total agreements divided by (total agreements + disagreements). The agreement was between 80% and 100% for all coding frame items, with an average agreement of 93.44%; which means the results are reliable.

## Results

### General Information

Direct-to-consumer telegenetics providers can be categorized into two general groups: commercial companies and non-commercial organizations (see Table 1). We identified six commercial consulting companies. Their websites generally had specialized designs. They also included comprehensive descriptions of their services and are aimed to advertise and sell genetic counseling and testing products. The remaining providers (*n* = 4) are non-commercial consulting organizations. Two of these were affiliated with universities; one with a private medical center; and one was offered by a government department (New York State Department of Health). Providers affiliated with non-commercial organizations usually did not have independent websites. The webpages of their services were commonly located under the official websites of the organizations and merely provided relatively simple instructions on how to access the services.

**Table 1:** DTC telegenetics providers.

Providers’ names	Websites	Locations
*Commercial companies*		
Generation Diagnostics	https://www.geneticstesting.com/telegenetic-counseling/	United States
Genetic Medicine Clinics	http://geneticmedicineclinic.com	United States
Genome Medical	https://www.genomemedical.com/	United States
Informed DNA	https://informeddna.com/	United States
Invitae	https://www.invitae.com/en/patients/resources/genetic-counseling/	United States
Telegenomics	https://telegenomics.com/	Spain
*Non-commercial organizations*		
Johns Hopkins Heart & Vascular Institute	https://www.hopkinsmedicine.org/heart_vascular_institute/clinical_services/centers_excellence/arvd/services/telegenetics.html	United States
New York State Department of Health, Wadsworth Center	https://www.wadsworth.org/programs/newborn/nymac/professionals/telegenetics	United States
OHSU Telemedicine Network	http://www.ohsu.edu/xd/health/for-healthcare-professionals/telemedicine-network/medical-services/tele-genetics-consultations.cfm	United States
Penn Medicine Abramson Cancer Centre	https://www.pennmedicine.org/for-patients-and-visitors/find-a-program-or-service/connected-care/virtual-consult/telegenetics	United States


Except for one Spanish company, all DTC telegenetics providers are U.S.-based. Seven providers described the concept of telegenetics on their webpages. The descriptions were offered in varying amounts of detail, but they all indicated that, by telephone or videoconference, professional genetic counselors were available to provide genetic counseling services. Two companies did not provide clear descriptions of telegenetics. One website provided a link to a 42-page document, where consumers could read an explanation of telegenetics and what to expect from the services. Seven providers indicated that their counseling services could lead to individually tailored treatments. Four commercial providers included claims that their counseling services involved genomic data analysis. In terms of target customers, two websites indicated that they serve only patients, and the rest (*n* = 8) target both patients and physicians. Three websites also provided services to commercial entities, such as firms and insurance companies.

### Services Provided

Twelve types of genetic and genomic services delivered through telegenetics were advertised directly to consumers via the providers’ websites (see Table 2). General genetic counseling and diagnosis of genetic diseases were offered on four and three websites, respectively. The providers tended to promote genetic services for specific diseases, with the top three being cancer genetics (advertised on seven websites), reproductive health genetics (advertised on five websites), and cardiac genetic counseling (advertised on four websites). On six websites providers used “best,” “optimal,” and “unique” to describe their services. Seven claimed that they will provide counseling services that integrate genetic testing results into the determination of health care interventions. For instance, as telegenomics claims on its website: “[w]e manage the genomic test most adequate to your needs, interpret the results and provide counseling for optimal treatment selection and prevention of the problems that may affect you or your family, today or in the future.”

**Table 2:** Frequency of services provided.

Services provided	Mentions
Cancer genetic services	7
Reproductive genetics	5
Genetic counseling about risks of diseases	4
Cardiac genetic counselor	4
Diagnosis of genetic diseases	3
Proactive health	3
Management of genetic diseases	2
Pharmacogenomics	1
Others	4


The costs of these services were shown on four out of ten websites. Generally, the cost for one appointment of telegenetics consultation ranged from 179 USD to 500 USD. One provider offered service packages, e.g., 179 USD per 30 min for proactive genetic exploration, and 250 USD for advanced genetic care. Another provider advised that if patients were covered by commercial health insurance, the typical coinsurance payment would be around 330 USD.

### Benefits and Risks

All the providers’ websites mentioned the benefits associated with telegenetics, while only one mentioned the potential risks (see Table 3). In total, we identified ten types of benefits advertised by providers. The most frequently claimed benefit is increased access to genetic counseling (all ten websites). Another frequently mentioned benefit is that the online services would provide timely and flexible hours for receiving genetic services (promoted on eight websites). Several other types of benefits mentioned targeted physicians and health plan companies. Among those were the importance of counseling by physicians in retaining patients (mentioned on four websites) and assisting health plan companies to craft better plans that cover only necessary genetic tests (mentioned on one website).

**Table 3:** Benefits and risks mentions.

Benefits	Mentions (%)
*Benefits for patients*	
Increased access to genetic counseling	10 (100)
Timely and flexible hours	8 (80)
Specialized consultation	7 (70)
Saving costs	4 (40)
Education on early detection	4 (40)
Community support among family members	1 (20)
Conquer barriers to medical care while providing the same high quality genetic counseling services	1 (20)
Patients are better informed to provide informed consent	1 (10)
*Benefits for physicians*	
Local providers can gain access services and information without referring their patients outside of their practice	4 (40)
*Benefits for health plan company*	
Significant savings for health plans by reducing inappropriate genetic testing requests	1 (10)
**Risks**	
Potential unauthorized access to consumers’ health information	1(10)


At the same time, risks associated with the implementation of telegenetics were most often not clearly mentioned. There was only one provider, i.e., John Hopkins Division of Cardiology, who indicated a potential risk in its informed consent form. In this form, the risk of unauthorized access to consumers’ health information during the course of telegenetic services was mentioned.

### Informed Consent

Only two websites provided potential clients with a link to an informed consent form. On one website, the Johns Hopkins Heart & Vascular Institute, the informed consent form could be easily found. The link to the informed consent form was shown on the main webpage. The document was quite shorter, i.e., less than one-page in length. It required clients to consent to disclose their protected health records. On the second website, Genetic Medicine Clinics, the informed consent form was hidden. The form offered a two-page document, which included a separate paragraph, addressing the release of a client’s personal medical records. According to this paragraph, by signing the form the consumer grants the provider with permission to release the personal medical records to public health agencies.

### Privacy Issues

With no exceptions, every website provided a link to privacy policy. Six providers offered a privacy document with a length of over three pages. Seven websites claimed that protective measures would be developed to minimize the risk of unauthorized access to clients’ personal data; three of which proposed concrete measures (see Table 4). One such measure was the training the staff to be more aware of the importance of privacy protection (mentioned on two websites). Another was employing a privacy officer who is responsible for developing, training and overseeing the implementation and enforcement of policies and procedures to safeguard clients’ personal health information (mentioned on one website). Every website included a statement that, in some circumstances, a client’s personal health information may be disclosed to public agencies according to federal or state laws.

**Table 4:** Concrete measures to protect the clients’ privacy.

Providers	Are concrete measures mentioned by the website to protect the clients’ privacy?	Examples of measures mentioned
Commercial companies	5 out of 6 mentioned	“This Site employs advanced technology (encryption provided through an SSL certificate) which aims to secure all of your interactions with INFORMED MEDICAL DECISIONS.” – https://informeddna.com/terms-conditions/ “We restrict access to your PHI to those workforce members who need access in order to provide services to our clients. We have established and maintain appropriate physical, electronic and procedural safeguards to protect your PHI against unauthorized use or disclosure. We train all workforce members on protecting your PHI. We also have a Privacy Officer, who has overall responsibility for developing, training and overseeing the implementation and enforcement of policies and procedures to safeguard your PHI against inappropriate access, use and disclosure.” – www.genomemedical.com/privacy/
Non-commercial organizations	2 out of 4 mentioned	“We will only use and disclose your personal health information (“PHI”) as allowed by law...We train our staff and work force to be sensitive about privacy and to respect the confidentiality of your PHI.” – https://www.pennmedicine.org/for-patients-and-visitors/patient-information/hipaa-and-privacy/hipaa-notice-of-privacy-practices


### The Use of Personal Health Data

Despite different wording, all websites addressed the use of personal health data, either in informed consent forms or privacy policy. Four websites stated that the company cannot sell a client’s information unless the client’s further permission has been obtained. Two companies indicated that they can reuse and share a client’s information for non-commercial purposes without the client’s further permission. Several providers differentiated identifiable and de-identified information and adopted different strategies for identifiable information. For instance, three companies indicated that the providers may reuse and share the identifiable health information without clients’ further permission for both commercial and non-commercial purposes, while two companies indicated that they will not sell or use the identifiable information to any other company or organization for direct marketing purposes.

## Discussion

Our study suggests that the DTC telegenetics industry, and especially telegenomics services, are still in their initial stages, with only four companies providing DTC telegenomics services identified through a Google search. While it is hard to estimate the future popularity of using DTC telegenetics for genetic and genomic counseling services, there are several practical indicators of its large potential. For example, such services are in line with the shift toward personalized and tailored medicine, which also fits into people’s tendency to focus on their personal needs and view themselves as unique individuals (Lyengar, 2010; Juengst et al., 2016). Additionally, the industry’s ability to employ information communication technology and geneticists networks will enable it to extend its services beyond geographic boundaries (Lea et al., 2005; Vrecar et al., 2017). This carries potential benefits for enhancing health communications, promoting awareness of genetic diseases, and improving professional support to consumers who access DTC genetic testing (Middleton et al., 2017). Moreover, the prices of these services – no more than a few hundred dollars – are likely to appeal to a wide range of consumers.

Our research has identified several legal and policy concerns that regulatory bodies should address urgently. In general, DTC telegenetics providers advertise genetic testing and related genomic research as an advanced, improved and revolutionary approach to health management. Existing literature has shown that DTC promotional materials tend to make inaccurate statements that may mislead consumers with regard to the clinical readiness of cutting-edge technologies, e.g., stem cells (Lau et al., 2008). Our study found a similar trend with respect to the advertising of telegenetics. Other than diagnosing genetic diseases, commercial telegenetics companies also provide risk assessment for common diseases, as well as recommend tailored treatments and proactive health advice based on their counseling services. For example, invitae states on its website that: “genetic insights can change everything.” In contrast, existing studies have noted that there is insufficient scientific evidence supporting the use of genomic profiles for assessing risks for common diseases and developing personalized health recommendations for preventing diseases (Janssens et al., 2008; Vayena, 2015; Khoury, 2017; Letai, 2017). In this regard, any claims that telegenetics will produce tailored health interventions and personalized medical treatments may mislead consumers about the clinical validity of genetic testing and genomics services (Niemiec et al., 2017).

Particularly, our study found that the portrayals of telegenetics on provider websites are imbalanced and extremely positive. The potential benefits of DTC telegenetics are presented in an absolute manner, using enticing terms such as “timely”, “flexible”, and “money saving”. In contrast, only one website mentioned the risks and limitations associated with the use of telemedicine in genetic services, addressing unintentional access to personal health information by an unauthorized third party and privacy breaches. Even if consumers are aware of the risks of misuse of personal health data and privacy concerns, providers do not afford patients sufficient and effective measures to protect their personal genomic information obtained through telegenetics. There is a growing concern about privacy protection in DTC genetic testing, and privacy worries are indeed a valid concern in the context of DTC telegenetics (Laestadius et al., 2017).

To our surprise, we did not find significant differences in the ways the commercial and non-commercial providers approached privacy. Our study revealed that privacy policies on half of the websites (*n* = 5) allow providers to use and share client’s health information with other entities, e.g., open-source databases, without seeking clients’ permission. Although two providers stated that they will not share or sell clients’ identifiable information, de-identified information does not eliminate the risk of potential privacy breaches. Relatives share a similar genome and re-identification of consumers’ genetic information that can be discovered through a relatively simple process (Asche et al., 2017). The recent Golden State Killer case in the United States also illustrates the feasibility of identifying someone’s genetic information based on the DNA sample of his or her relative through an open-source genetic database (Scutti, 2018).

Another related risk is the ease with which it may be possible to send a third party’s sample for testing (May, 2018). Safety measures should ensure that providers strictly confirm their clients’ identity. The problem of online identity verification and authentication is relevant to various online domains, with one example being online dating sites. Not surprisingly then, there is a variety of techniques to address this challenge. For instance, providers may compare individual input (personal information) to available records from government agencies; require online customers to use a code sent to them by text, or to supply a copy of an identity card or a driving license; or detail information regarding previous interactions the customer had with the provider. Where circumstances allow, firms may use biometric techniques such as fingerprints, a voiceprint or a retina scan.

Most of the DTC telegenetics providers (nine out of ten) are based in the United States, where the *Health Insurance Portability and Accountability Act* [HIPAA] (1996), governs and regulates both disclosure and use of protected health information (Sfikas, 2002). However, de-identified information is not governed by the Standards for *Privacy of Individually Identifiable Health Information* (2000) (Privacy Rule), established by the United States Department of Health and Human Services (Standards for *Privacy of Individually Identifiable Health Information*, 2000; Thorpe and Gray, 2015; Asche et al., 2017). Therefore, even if the telegenetics providers promise not to share and sell identifiable personal health information including genetic information according to the *Privacy Rule*, clients’ health privacy may not be fully safeguarded by the *Privacy Rule* due to the risk of re-identification.

Furthermore, our study shows that several DTC telegenetics providers offer genetic and genomic services not only to patients and physicians but also to companies and insurance companies. Without efficient and effective frameworks, such services might enable companies to easily retrieve clients’ or their relatives’ personal health information, utilizing datasets collected from patients, physicians and employers (Shen and Ma, 2017). The increased possibility of attacking and de-identifying personal genetic data would then result in higher risks of genetic discrimination in the context of insurance. Take, for instance, the *Genetic Information Nondiscrimination Act* (2008). (GINA), which was established to prevent insurance companies from requesting individuals’ genetic information. This Act only applies to health insurance, but not to other types of insurance - including life and disability insurances (Parkman et al., 2015). Therefore, without an appropriate regulatory framework, the development of DTC telegenetics may impact consumers’ access to insurance and may raise legitimate fairness concerns in insurance markets.

Since anyone can access the genomics services via the internet, data privacy and security face an increasing risk of breaches when cross-border data flows and transfers occur. It is noteworthy that several transnational agreements have been developed to address the protection of personal data protection. For example, in 2016, the United States Department of Commerce and the European Commission developed a new framework, the EU-US Privacy Shield (2018). However, the framework is not mandatory for a United States company and all telegenetics providers’ websites that we examined have not joined the framework. This entails that privacy concerns are aggravated for international telegenetic services since no stringent regime for data privacy protection has been established at the transnational level.

## Limitations

This study does not represent the whole of online genetic counseling and testing services offered through telemedicine technology. Rather, it examines the websites of providers that focus on, and primarily offer, DTC telegenetics or telegenomics services. Such services reflect a very recent development in the market of specialized genetic counseling services. While this focus produced unique and important findings, the sample size is nevertheless small.

Moreover, the study does not examine consumers’ perspective. Future empirical research should examine how well consumers understand the risks involved in DTC telegenetics and telegenomics services. It should also inquire into the way consumers view the informed consent process. In particular, we suggest that further attention will be given to consumers’ ability to become informed via alternative platforms and channels of communication, including social media, peer-to-peer information flows, tutorials and the like.

## Conclusion

Direct-to-consumer telegenetics can increase patients’ knowledge of genetic diseases and their access to genetic and genomics services. Where traditional genomic services are not widely available, DTC telegenetics may improve local genetic services and medical care treatments and lower costs. Commercial providers identified the business potential in this field and started using genomic data to offer a range of services.

However, before DTC telegenetics can flourish at a global scale, more research and regulatory improvements are required. Our findings indicate that the genetic and genomic services offered through telemedicine were portrayed extremely positively by DTC telegenetics providers. At the same time, risks associated with the implementation of telegenetics were rarely mentioned. By providing an unrealistic rosy perspective, these websites can exploit and exacerbate consumers’ over-optimism.

On top of that, consent forms are often not conspicuous, and they do not contain all the relevant information. Given the way firms misrepresent their services, it may be impossible to obtain meaningful consent to begin with. If consumers are unaware of the true nature and the risks of the service being offered, it is doubtful whether they are able to genuinely consent to it.

Likewise, measures for protecting clients’ personal health information by telegenetics providers are generally inadequate and weak. Providers are now offering not only diagnostic services but also risk assessments for common diseases and personalized lifestyle recommendations. However, relevant U.S. laws may fail to provide sufficient protection for clients’ health data, which may lead to privacy breaches and unjust discrimination. This is especially troubling in light of the fact that genetic services involve sensitive information that is at the core of one’s identity.

In light of the unique nature and context of the telegenomics market, regulatory steps require a nuanced analysis and a multidisciplinary perspective. This study indicates that special attention should be directed towards: truthful advertising and scientifically accurate presentation of these services; obtaining informed consent; securing personal health data sharing; and generally guaranteeing adequate privacy protection.

Future studies should examine whether current rules and laws regarding advertising – such as the ones introduced by the Federal Drug Administration and the Federal Trade Commission – can provide a useful framework for regulating the presentations made by DTC telegenetics and telegenomics services. To meet the needs of a rapidly growing and dynamic industry, it seems advisable to periodically examine the proper legal and policy framework (Becher, 2018). Currently, consumers are left without proper protection of some of their most valuable interests and rights.

## Author Contributions

LD and SB designed the study. LD collected and analyzed the data and drafted the manuscript. SB made substantial revisions to the manuscript.

## Conflict of Interest Statement

The authors declare that the research was conducted in the absence of any commercial or financial relationships that could be construed as a potential conflict of interest.
